# Supplementary material for: Permian hypercarnivore suggests dental complexity among early amniotes
Source: Nat Commun. 2022 Aug 19;13:4882. doi: 10.1038/s41467-022-32621-5 (PMC9391490; doi:10.1038/s41467-022-32621-5)
Supplement: Supplementary file 1 — Supplementary Information [file 41467_2022_32621_MOESM1_ESM.pdf]

## Supplementary Figures and Table

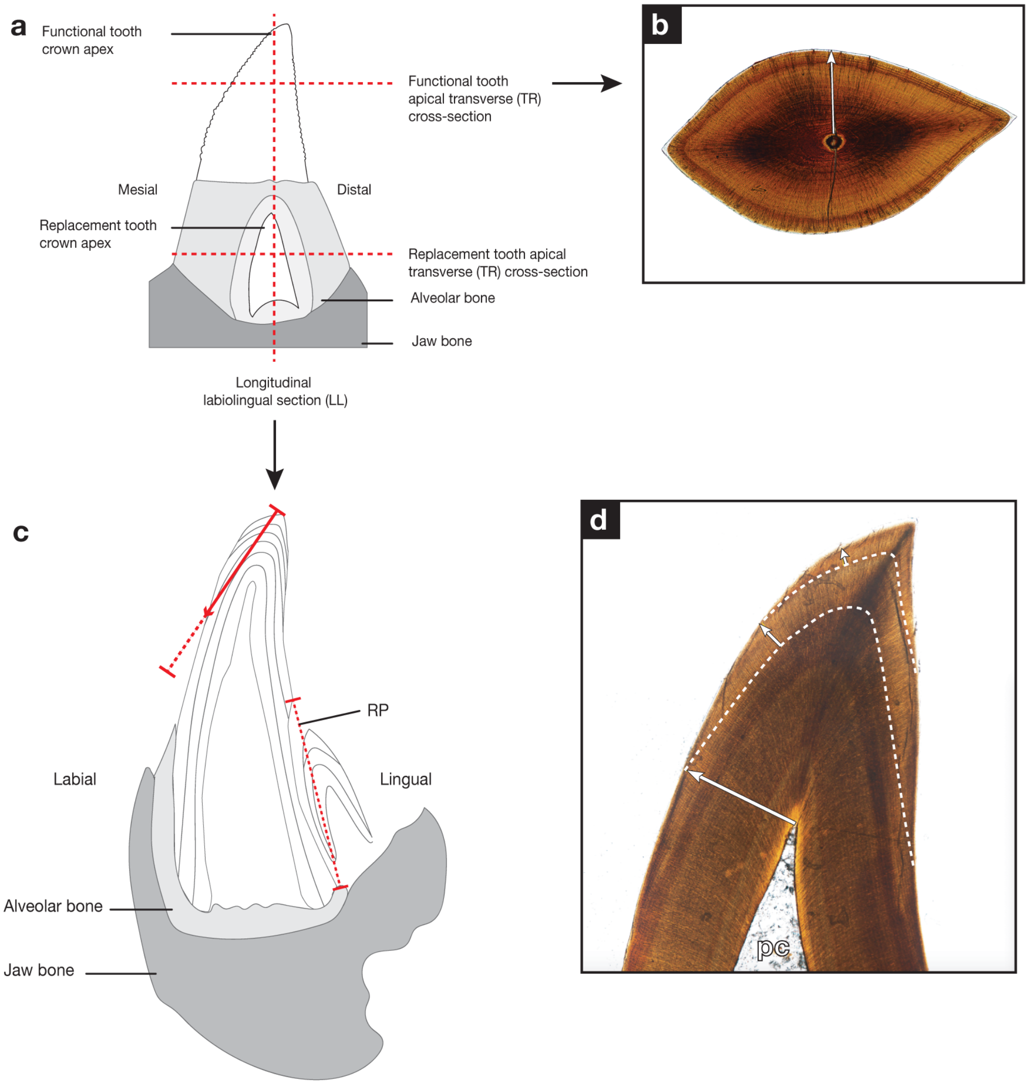


**Supplementary Figure 1. Theoretical tooth family for incremental growth line counts**. (a) drawing of tooth family in lingual view, showing functional tooth, replacement tooth, plane of longitudinal labiolingual (LL) section, and plane of transverse sections. (b) transverse section of ROMVP 85445 functional tooth showing incremental line trajectories; counts made from pulp cavity to the outer edge of the dentine, perpendicular to incremental line trajectories (white arrow). (c) drawing of tooth family in LL section, showing the functional tooth and the replacement tooth, with the estimated height of missing replacement tooth shown as a solid red line, while the dashed red line is the total height of resorption pit. (d) LL section of ROMVP 85455 functional tooth showing incremental line trajectories; counts are made from the pulp cavity to the outer edge of the dentine (white dotted line), perpendicular to incremental line trajectories. Abbreviations: RP, resorption pit.

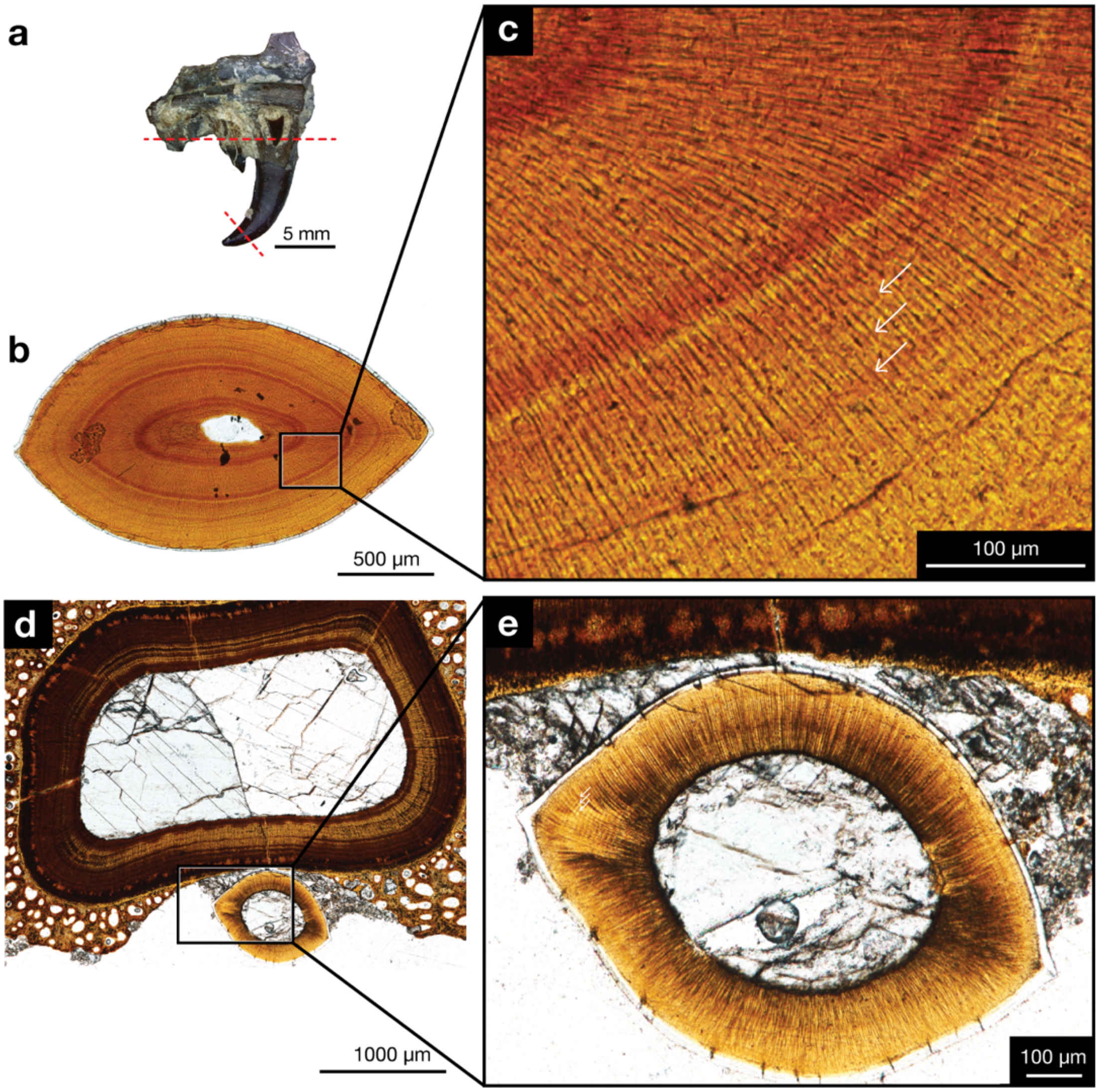


**Supplementary Figure 2. Incremental lines of *Mesenosaurus efremovi.*** (a) ROMVP 85443, lingual view of maxilla with dashed red lines through the planes of transverse sections of the functional and replacement teeth. (b) whole view of functional tooth transverse SC near crown apex. (c) closeup view of functional tooth TR cross-section showing incremental lines, white arrows. (d) whole view of the functional tooth and replacement tooth TR cross-section near crown apex of replacement tooth(e) closeup view of replacement tooth TR cross-section showing incremental lines, white arrows.

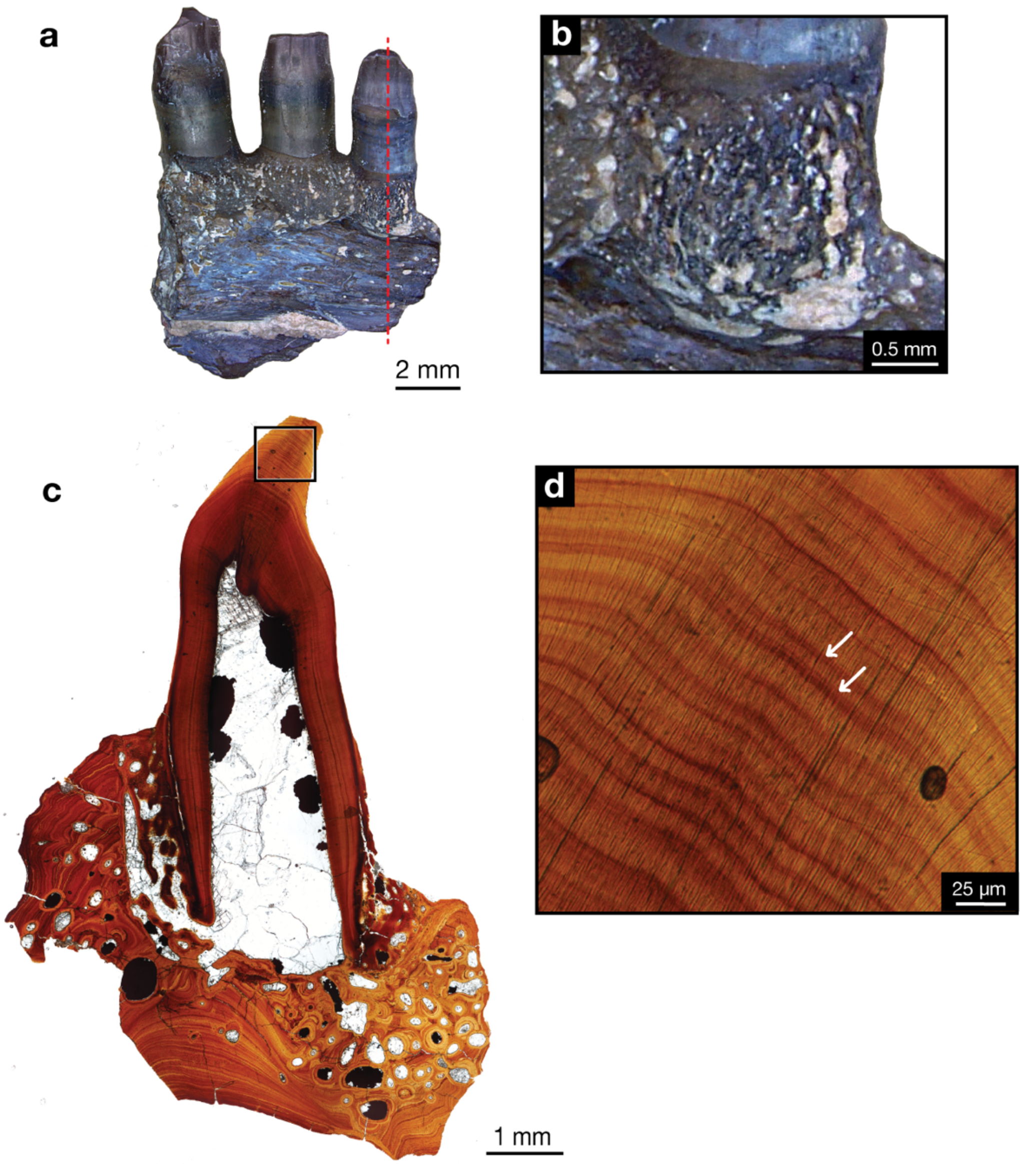


**Supplementary Figure 3. Incremental lines of *Oromycter.*** (a) ROMVP 85516, lingual view of maxilla showing with dashed red lines the plane of longitudinal LL sections through functional tooth (mx07) with a resorption pit. (b) close-up of resorption pit on the tooth. (c) whole view of functional tooth section near the crown apex. (d) close-up view of functional tooth section showing incremental lines, white arrows.

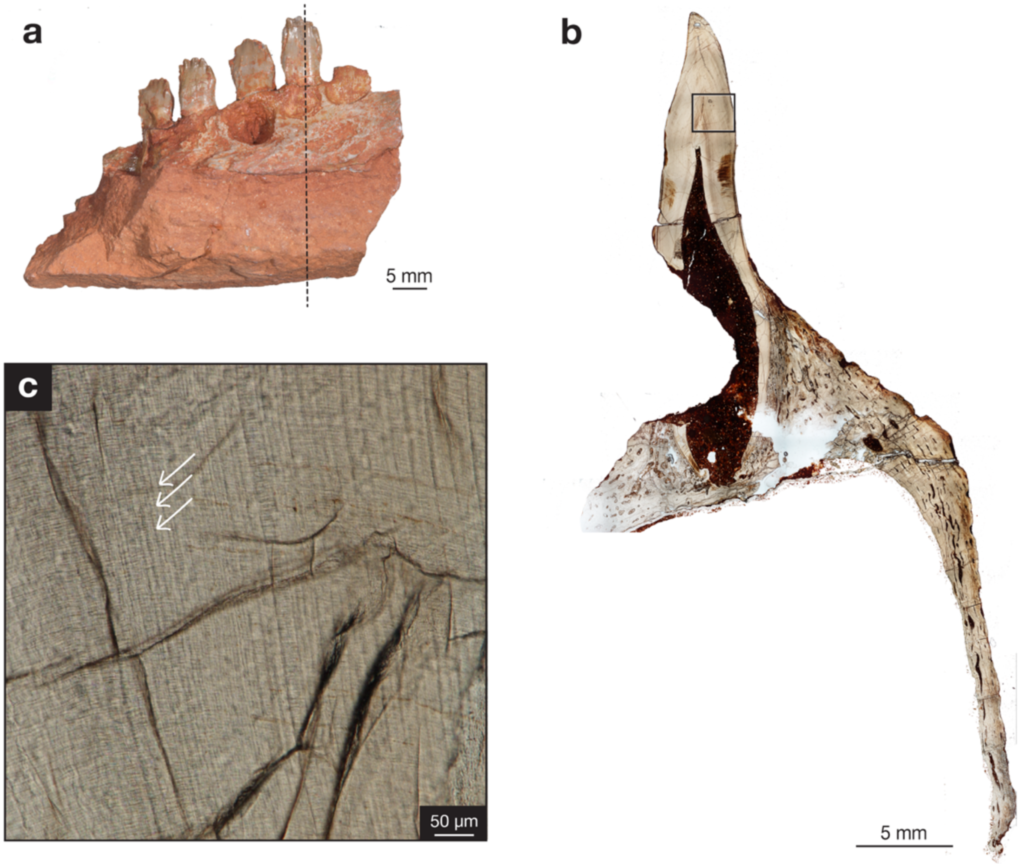


**Supplementary Figure 4. Incremental lines of *Ennatosaurus tecton*.** (a) PIN 4543, lingual view of dentary with a dashed black line through the plane of longitudinal LL section of the functional tooth (d08) with a resorption pit. (b) whole view of functional tooth section near the crown apex. (c) closeup view of functional tooth section showing incremental lines, white arrows.

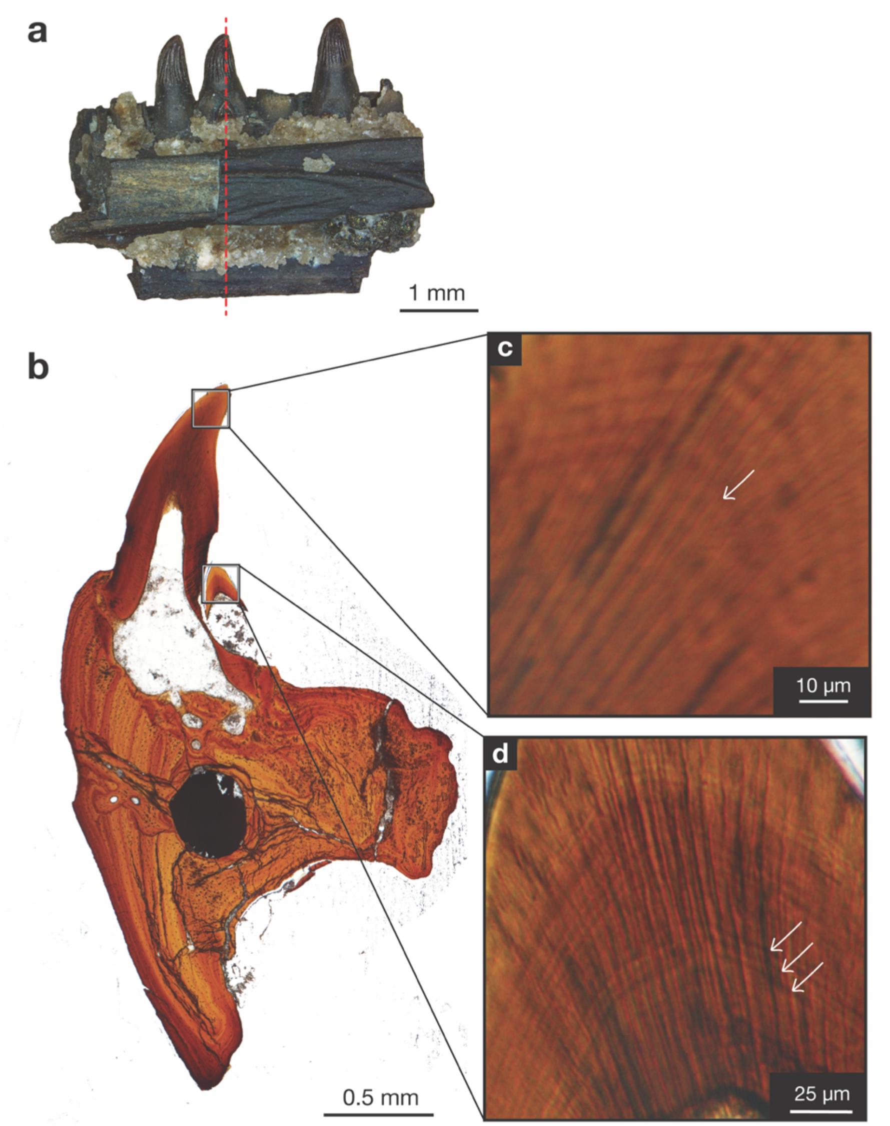


**Supplementary Figure 5. Incremental lines of *Delorhynchus*.** (a) ROMVP 85512, lingual view of dentary showing with dashed red lines the plane of LL section through functional and replacement teeth. (b) whole view of functional tooth longitudinal LL near the crown apex. (c) closeup view of functional tooth LL cross-section showing incremental lines, white arrows. (d) closeup view of replacement tooth LL cross-section showing incremental lines, white arrows.

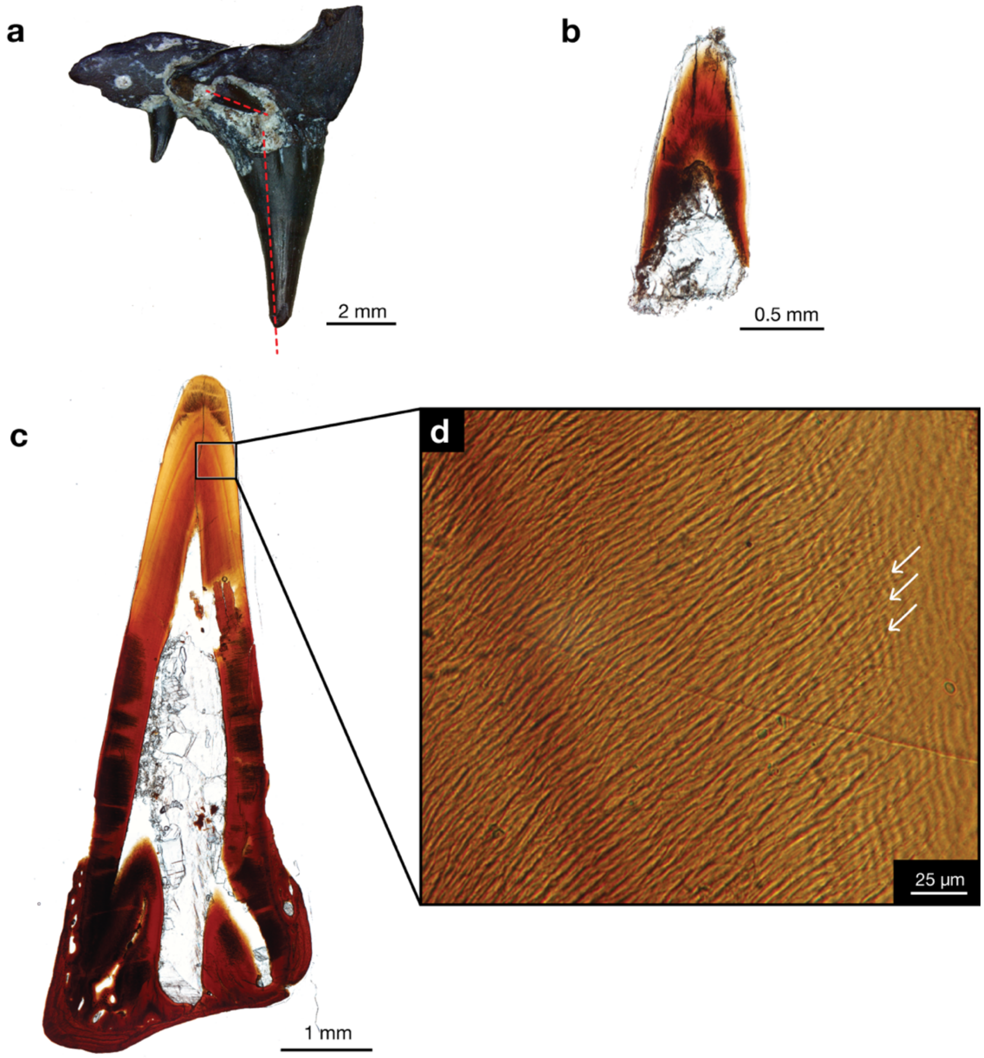


**Supplementary Figure 6. Incremental lines of *Colobomycter*.** (a) ROMVP 85521, lingual view of premaxilla showing with dashed red lines for the plane of LL sections through functional and replacement teeth. (b) whole view of replacement tooth longitudinal LL near the crown apex. (c) whole view of functional tooth longitudinal LL near the crown apex. (d) closeup view of functional tooth LL cross-section showing incremental lines, white arrows.

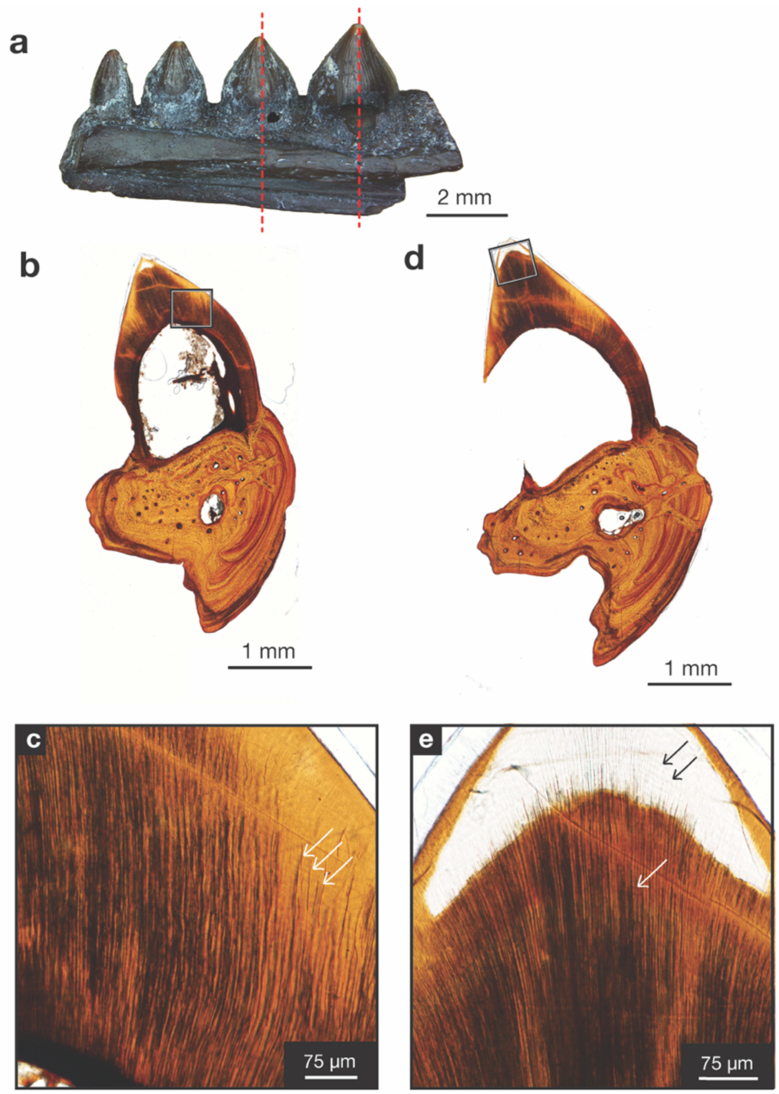


**Supplementary Figure 7. Incremental lines of *Opisthodontosaurus.*** (a) ROMVP 85511, lingual view of dentary showing with dashed red lines the plane of LL section through two functional teeth with resorption pits. (b) whole view of d06 tooth LL section near the crown apex. (c) closeup view of d06 tooth LL cross-section showing incremental lines, white arrows. (d) whole view of d07 tooth LL cross-section near the crown apex of the replacement tooth. (e) closeup view of replacement d07 tooth LL cross-section showing incremental lines, white and black arrows.

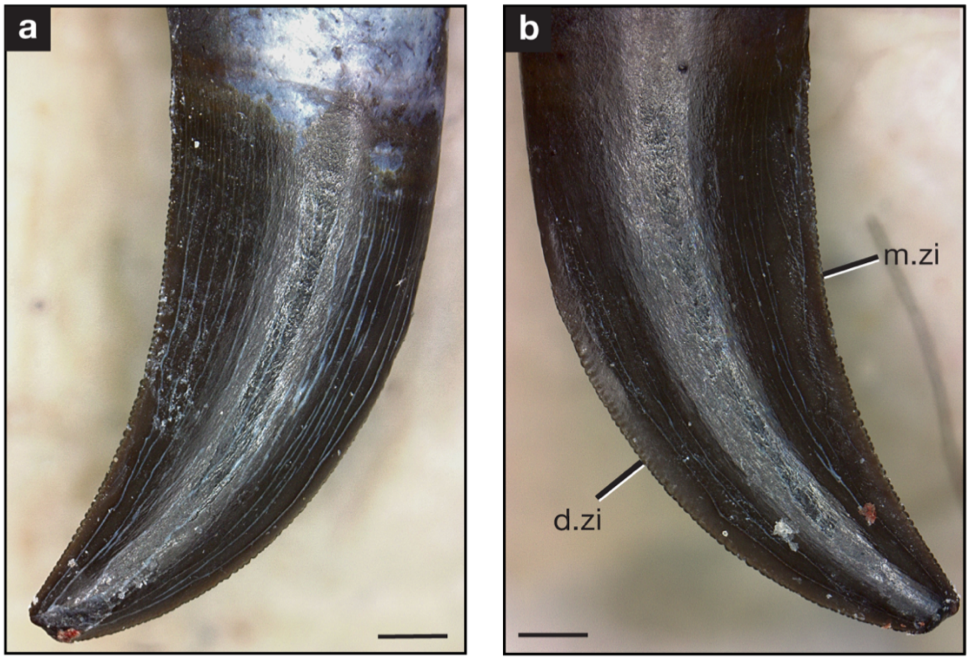


**Supplementary Figure 8. Serrations on tooth crown of *Mesenosaurus*.** ROMVP 85469 (a) labial and (b) lingual view of maxillary tooth crown. Abbreviations: d.zi, distal ziphodonty; m.zi, mesial ziphodonty. Scale = 500 µm.

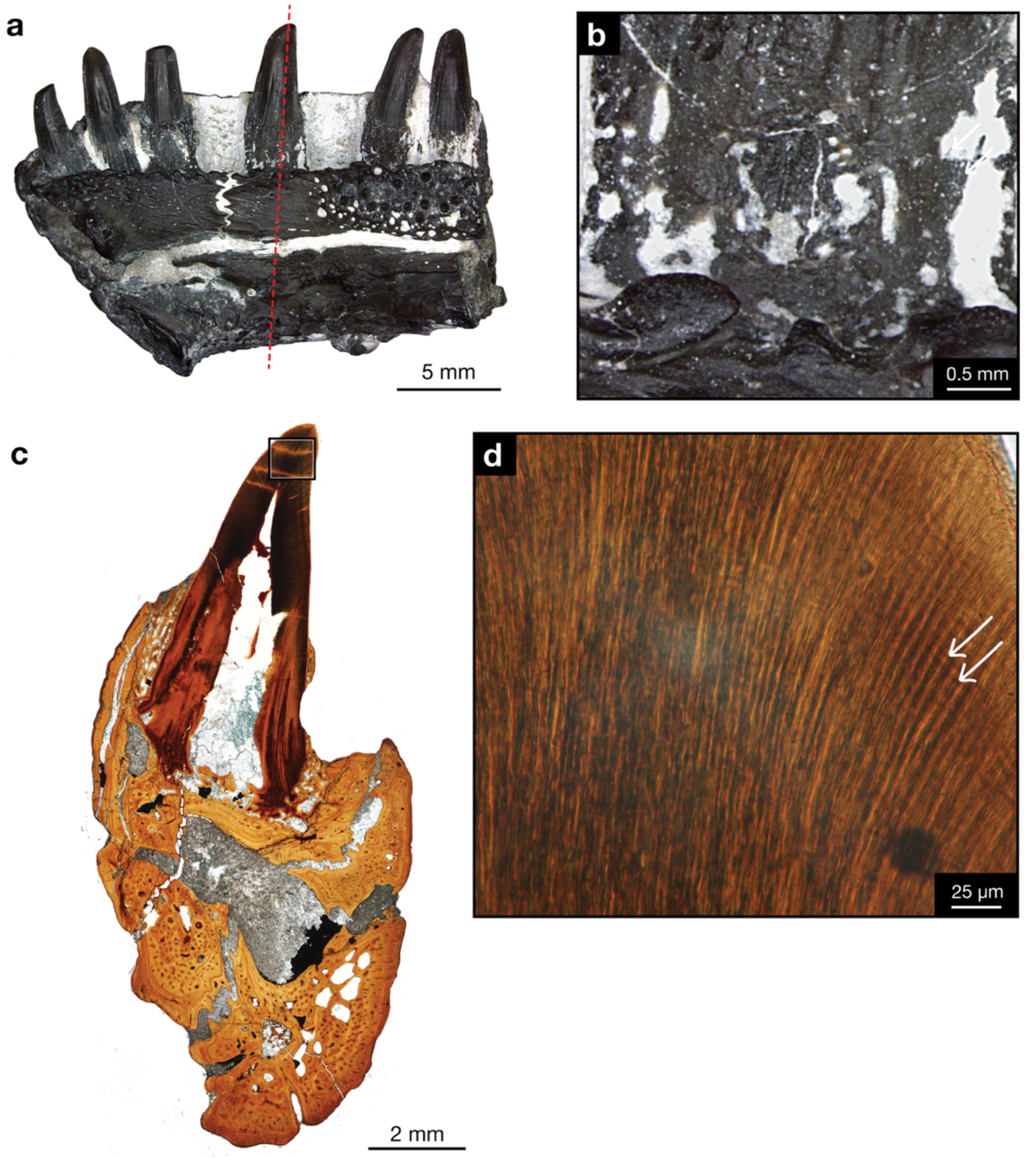


**Supplementary Figure 9. Incremental lines of *Seymouria.*** (a) ROMVP 85515, lingual view of dentary with a dashed red line through the plane of longitudinal LL section of the functional tooth (d05) with a resorption pit. (b) close-up of resorption pit on the tooth. (c) whole view of functional tooth section near the crown apex. (d) close-up view of functional tooth section showing incremental lines, white arrows.

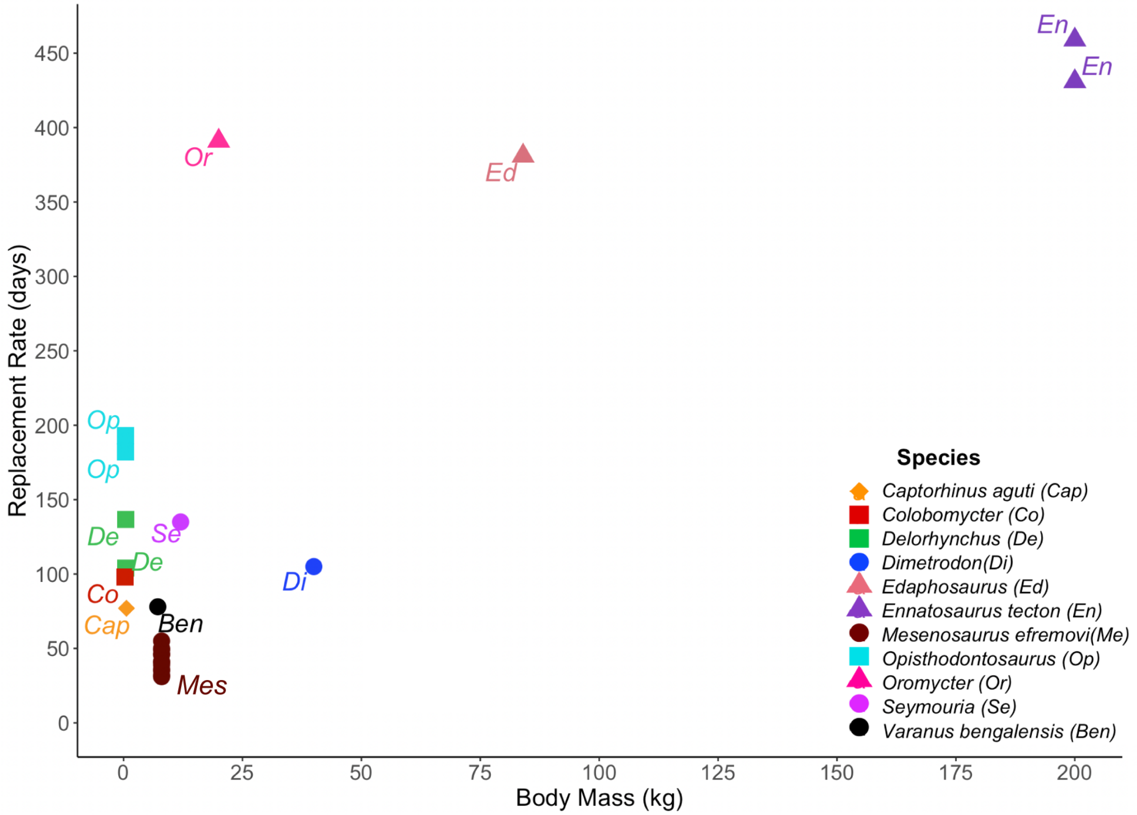


**Supplementary Figure 10. Rates of tooth replacement and body mass across a range of taxa.** Relationship between the estimated body mass (kg) and tooth families replacement rate (days). The symbols indicate the type of feeding behaviour, with circles representing carnivory, triangles representing herbivory, square representing insectivory, and diamond representing omnivory. Source data are provided as a Source Data file.

**Table 1.** Total number of incremental von Ebner lines and mean incremental line width (µm) for *Mesenosaurus efermovi (Mes)*, *Dimetrodon* cf *(Dim)*. *limbatus, Haptodus (Hap), Watongia meieri (Wat)*, *Oromycter (Oro), Edaphosaurus* sp. *(Eda)*, *Ennatosaurus tecton (Enn), Delorhynchus (Del), Opisthodontosaurus (Opi)*, *Captorhinus (Cap)*, *Colobomycter (Col)*, *Seymouria (Sey)*, *Varanus bengalensis (Ben),* and *Varanus komodoensis* (*Kom)* using AP, TR, and LL sections. The approximate sign (~) before the replacement tooth implies that the age was estimated for the missing replacement tooth using the method described above.

| **Taxon code** | **Specimen** | **Total number of incremental lines** | | **Mean incremental line width (µm)** | | **Functional tooth height (mm)** | **Type of Section** |
|---|---|---|---|---|---|---|---|
| | | **Functional tooth** | **Replacement tooth** | **Functional tooth** | **Replacement tooth** | | |
| *Mes* | ROMVP 85502 | 71 | 32 | 5.41 ± 1.4 | 3.16 ± 1.07 | 3.42 | LL |
| *Mes* | ROMVP 85503 | 74 | 25 | 6.51 ± 2.53 | 5.46 ± 1.46 | 3.29 | LL |
| *Mes* | ROMVP 85504 | 89 | 57 | 6.87 ± 1.28 | 6.09 ± 1.2 | 4.15 | LL |
| *Mes* | ROMVP 85507 | 102 | 61 | 4.07 ± 0.69 | 4.52 ± 0.68 | 3.21 | LL |
| *Mes* | ROMVP 85506 | 83 | 28 | 3.53 ± 0.66 | 4.58 ± 1.22 | 1.44 | LL |
| *Mes* | ROMVP 85505 | 86 | 45 | 3.74 ± 0.84 | 2.37 ± 0.38 | 3.09 | LL |
| *Mes* | ROMVP 85508 | 62 | 31 | 3.8 ± 0.8 | 3.29 ± 0.74 | 1.63 | LL |
| *Mes* | ROMVP 85509 | 73 | 23 | 5.47 ± 2.17 | 4.55 ± 0.89 | 2.32 | LL |
| *Mes* | ROMVP 85457-T1 | 64 | 29 | 7.06 ± 2.21 | 7.15 ± 1.39 | 2.82 | LL |
| *Mes* | ROMVP 85457-T2 | 59 | 23 | 6.85 ± 1.25 | 7.43 ± 1.23 | 4.19 | LL |
| *Mes* | ROMVP 85457-T3 | 72 | 26 | 6.20 ± 1.83 | 3.91 ± 0.76 | 4.18 | LL |
| *Mes* | ROMVP 85491 | 92 | 46 | 4.08 ± 1.5 | 2.2 ± 0.39 | 3.28 | LL |
| *Mes* | ROMVP 85455 | 94 | 64 | 6.85 ± 1.25 | 4.93 ± 0.82 | 3.64 | LL |
| *Mes* | ROMVP 85445 | 68 | 25 | 3.25 ± 0.83 | 3.91 ± 1.23 | 2.45 | TR |
| *Mes* | ROMVP 85443 | 56 | 22 | 9.08 ± 0.85 | 5.53 ± 0.81 | 4.67 | TR |
| *Dim* | ROMVP 85510 | 459 | 354 | 5.46 ± 1.2 | 2.68 ± 0.83 | 15.34 | LL |
| *Wat* | UCMP 143278-001-T1 | 81 | - | 10.32 ± 1.87 | - | 5.99 | LL |
| *Wat* | UCMP 143278-001-T2 | 68 | - | 11.88 ± 1.73 | - | 6.48 | LL |
| *Wat* | UCMP 143278-002 | 145 | - | 7.99 ± 2.5 | - | 8.13 | LL |
| *Wat* | UCMP 143278-003 | 108 | - | 15.89 ± 3.89 | - | 9.79 | LL |
| *Oro* | ROMVP 85516-T1 | 426 | - | 4.24 ± 1.06 | - | 5.96 | LL |
| *Oro* | ROMVP 85516-T2 | 506 | ~115 | 3.92 ± 0.79 | - | 4.42 | LL |
| *Enn* | PIN 4543-T1 | 567 | ~136 | 3.69 ± 1.13 | - | 11.30 | LL |
| *Enn* | PIN 4543-T2 | 628 | ~169 | 2.82 ± 1.2 | - | 9.96 | LL |
| *Enn* | PIN 4543-T3 | 505 | - | 3.17 ± 1.13 | - | 9.57 | LL |
| *Eda* | USNM PAL 706602-T1 | 429 | - | 4.29 ± 1.98 | - | 12.27 | LL |
| *Eda* | USNM PAL 706602-T2 | 506 | ~131 | 4.2 ± 1.29 | - | 12.89 | AP |
| *Del* | ROMVP 85512 | 147 | 43 | 2.38 ± 0.32 | 1.69 ± 0.22 | 0.84 | LL |

| | | | | | | | |
|---|---|---|---|---|---|---|---|
| *Del* | ROMVP 85513 | 169.48 | 32.82 | 3.06 ± 0.45 | 3.06 ± 0.45 | 1.84 | LL |
| *Del* | ROMVP 85514-T1 | 169 | - | 2.917 ± 0.8 | - | 2.12 | LL |
| *Del* | ROMVP 85514-T2 | 152 | - | 3.00 ± 0.78 | - | 2.10 | LL |
| *Col* | ROMVP 85521 | 157 | 59 | 3.65 ± 0.92 | 3.47 ± 0.28 | 5.19 | LL |
| *Opi* | ROMVP 85511-T1 | 151 | - | 1.91 ± 0.46 | - | 1.74 | LL |
| *Opi* | ROMVP 85511-T2 | 155 | - | 3.22 ± 0.66 | - | 2.05 | LL |
| *Opi* | ROMVP 85511-T3 | 206 | ~24 | 2.65 ± 1.03 | - | 2.26 | LL |
| *Opi* | ROMVP 85511-T4 | 258 | ~65 | 2.01 ± 0.46 | - | 2.86 | LL |
| *Opi* | ROMVP 85526-T1 | 154 | - | 3.68 ± 0.87 | - | 2.43 | AP |
| *Opi* | ROMVP 85526-T2 | 113 | - | 3.77 ± 1.07 | - | 2.31 | AP |
| *Opi* | ROMVP 85527-T1 | 189 | - | 2.99 ± 0.65 | - | 1.92 | AP |
| *Opi* | ROMVP 85527-T2 | 134 | - | 2.44 ± 0.52 | - | 1.64 | AP |
| *Cap* | ROMVP 85525 | 146 | 69 | 3.04 ± 0.62 | 5.14 ± 0.94 | 3.69 | LL |
| *Sey* | ROMVP 85515-T1 | 126 | - | 5.22 ± 0.76 | - | 4.73 | LL |
| *Sey* | ROMVP 85515-T2 | 171 | ~36 | 6.71 ± 0.92 | - | 4.29 | LL |
| *Ben* | ROM R271 | 188 | ~78 | 5.15 ± 1.28 | 5.15 ± 1.28 | 2.64 | LL |
| *Kom* | ROM R10117 | 157.72 | - | 8.35 ± 0.87 | - | 13.12 | LL |
| *Kom* | ROM R7565-T1 | 104.51 | - | 9.05 ± 1.09 | - | 11.32 | LL |
| *Kom* | ROM R7565-T2 | 105.89 | - | 7.98 ± 1.96 | - | 12.96 | LL |
| *Kom* | ROM37036 | 152 | - | 8.24 ± 1.75 | - | 5.09 | LL |
| *Hap* | *Hap*-UTM-001 | 151.28 | - | 3.41 ± 0.49 | - | 2.46 | LL |
