# Supplementary material for: Permian hypercarnivore suggests dental complexity among early amniotes
Source: Nat Commun. 2022 Aug 19;13:4882. doi: 10.1038/s41467-022-32621-5 (PMC9391490; doi:10.1038/s41467-022-32621-5)
Supplement: Supplementary file 3 — Reporting Summary [file 41467_2022_32621_MOESM3_ESM.pdf]

nature portfolio

Corresponding author(s): Professor Robert Reisz

Last updated by author(s): Jul 4, 2022

Reporting Summary

Nature Portfolio wishes to improve the reproducibility of the work that we publish. This form provides structure for consistency and transparency in reporting. For further information on Nature Portfolio policies, see our Editorial Policies and the Editorial Policy Checklist.

Statistics

For all statistical analyses, confirm that the following items are present in the figure legend, table legend, main text, or Methods section.

n/a	Confirmed	
☐	☒	The exact sample size (*n*) for each experimental group/condition, given as a discrete number and unit of measurement
☒	☐	A statement on whether measurements were taken from distinct samples or whether the same sample was measured repeatedly
☒	☐	The statistical test(s) used AND whether they are one- or two-sided<br>*Only common tests should be described solely by name; describe more complex techniques in the Methods section.*
☒	☐	A description of all covariates tested
☒	☐	A description of any assumptions or corrections, such as tests of normality and adjustment for multiple comparisons
☐	☒	A full description of the statistical parameters including central tendency (e.g. means) or other basic estimates (e.g. regression coefficient) AND variation (e.g. standard deviation) or associated estimates of uncertainty (e.g. confidence intervals)
☒	☐	For null hypothesis testing, the test statistic (e.g. *F*, *t*, *r*) with confidence intervals, effect sizes, degrees of freedom and *P* value noted<br>*Give P values as exact values whenever suitable.*
☒	☐	For Bayesian analysis, information on the choice of priors and Markov chain Monte Carlo settings
☒	☐	For hierarchical and complex designs, identification of the appropriate level for tests and full reporting of outcomes
☒	☐	Estimates of effect sizes (e.g. Cohen's *d*, Pearson's *r*), indicating how they were calculated

Our web collection on statistics for biologists contains articles on many of the points above.

Software and code

Policy information about availability of computer code

Data collection	No software was used for data collection
Data analysis	Image J version 2.0.0 and R studio version 2021.09.02

For manuscripts utilizing custom algorithms or software that are central to the research but not yet described in published literature, software must be made available to editors and reviewers. We strongly encourage code deposition in a community repository (e.g. GitHub). See the Nature Portfolio guidelines for submitting code & software for further information.

Data

Policy information about availability of data

All manuscripts must include a data availability statement. This statement should provide the following information, where applicable:

- Accession codes, unique identifiers, or web links for publicly available datasets
- A description of any restrictions on data availability
- For clinical datasets or third party data, please ensure that the statement adheres to our policy

The data that support the findings of this study are available from the corresponding author, RRR, and have been uploaded to Morphobank

Field-specific reporting

Ecological, evolutionary & environmental sciences study design

All studies must disclose on these points even when the disclosure is negative.

Study description	Comparing dental developmental rates and function of teeth across nearly 300 million years of evolution between an early amniote predator and the spectacular living Komodo dragon in order to draw parallels.
Research sample	Early Permian taxa: Mesenosaurus efremovi, Oromycter, Watongia meieri, Dimetrodon cf. D.limbatus, Haptodus sp., Edaphosaurus sp., Ennatosaurus tecton, Opisthodontosaurus, Captorhinus, Delorhynchus, Colobomycter, and Seymouria.<br>Extant varanid taxa: Varanus komodoensis and Varanus bengalensis. Samples are very difficult to acquire. They were chosen on the basis of phylogenetic and temporal proximity to the species being studied, with comparisons with extant taxa that exhibited similar morphological characteristics.
Sampling strategy	Fossil materials for destructive sampling are very difficult to acquire. In the case of all specimens, where available multiple loci of the dentigerous elements were sampled. Sample sizes were sufficient for the type of research conducted in this study
Data collection	The tooth longevity data were recorded by TM by counting the lines of von Ebner within the dentine for the histological sections. The replacement rate data was calculated by TM by subtracting the replacement tooth age from the functional tooth age.
Timing and spatial scale	Since these are fossils timing of data collection was not relevant. Extra effort was taken in selecting taxa that are approximately the same age.
Data exclusions	No data were excluded.
Reproducibility	The data generated by this study are original observations not subject to reprodudibility. They can be replicated if need by any future study, but new specimens would have to be found, a very difficult task.
Randomization	Randomization was not required since the study and the sample size do not lend themselves to such an appraoch
Blinding	Blinding was not relevant for our study since we were examining the tooth development and replacement for early Permian taxa that have not been studied before in this matter and no assumptions were made prior.

Did the study involve field work? ☐ Yes ☒ No

Reporting for specific materials, systems and methods

We require information from authors about some types of materials, experimental systems and methods used in many studies. Here, indicate whether each material, system or method listed is relevant to your study. If you are not sure if a list item applies to your research, read the appropriate section before selecting a response.

Materials & experimental systems

n/a	Involved in the study	
☒	☐	Antibodies
☒	☐	Eukaryotic cell lines
☐	☒	Palaeontology and archaeology
☒	☐	Animals and other organisms
☒	☐	Human research participants
☒	☐	Clinical data
☒	☐	Dual use research of concern

Methods

n/a	Involved in the study	
☒	☐	ChIP-seq
☒	☐	Flow cytometry
☒	☐	MRI-based neuroimaging

Palaeontology and Archaeology

Specimen provenance	Specimens were donated to the senior author by a private collector Mr. Bill May, who received verbal permission from the owners of the properties where they originated. That is all that is required in the case of small fossil samples used in this study
Specimen deposition	Royal Ontario Museum (ROM)
Dating methods	The ages of the fossils used in this study have been determined on the basis of previous publications, and age determination is not part of this research

☐ Tick this box to confirm that the raw and calibrated dates are available in the paper or in Supplementary Information.

Ethics oversight	The fossil materials are more than 250 million years old and do not required ethical oversight. No excavation program was involved, and if not collected, these materials would have been destroyed by natural erosion.

Note that full information on the approval of the study protocol must also be provided in the manuscript.
